# Electrocatalytic NAD^+^ reduction *via* hydrogen atom-coupled electron transfer†

**DOI:** 10.1039/d2sc02691k

**Published:** 2022-10-24

**Authors:** Fengyuan Liu, Chunmei Ding, Shujie Tian, Sheng-Mei Lu, Chengcheng Feng, Dandan Tu, Yan Liu, Wangyin Wang, Can Li

**Affiliations:** Zhang Dayu School of Chemistry, Dalian University of Technology Dalian 116024 Liaoning China; State Key Laboratory of Catalysis, Dalian Institute of Chemical Physics, Chinese Academy of Sciences, Dalian National Laboratory for Clean Energy Dalian 116023 China canli@dicp.ac.cn; University of Chinese Academy of Sciences Beijing 100049 China; School of Chemistry and Materials Science, University of Science and Technology of China Hefei 230026 China

## Abstract

Nicotinamide adenine dinucleotide cofactor (NAD(P)H) is regarded as an important energy carrier and charge transfer mediator. Enzyme-catalyzed NADPH production in natural photosynthesis proceeds *via* a hydride transfer mechanism. Selective and effective regeneration of NAD(P)H from its oxidized form by artificial catalysts remains challenging due to the formation of byproducts. Herein, electrocatalytic NADH regeneration and the reaction mechanism on metal and carbon electrodes are studied. We find that the selectivity of bioactive 1,4-NADH is relatively high on Cu, Fe, and Co electrodes without forming commonly reported NAD_2_ byproducts. In contrast, more NAD_2_ side product is formed with the carbon electrode. ADP-ribose is confirmed to be a side product caused by the fragmentation reaction of NAD^+^. Based on H/D isotope effects and electron paramagnetic resonance analysis, it is proposed that the formation of NADH on these metal electrodes proceeds *via* a hydrogen atom-coupled electron transfer (H_ad_CET) mechanism, in contrast to the direct electron-transfer and NAD˙ radical pathway on carbon electrodes, which leads to more by-product, NAD_2_. This work sheds light on the mechanism of electrocatalytic NADH regeneration, which is different from biocatalysis.

## Abstract 2

A hydrogen atom coupled with electron transfer (H_ad_CET) mechanism is proposed for electrocatalytic NAD^+^ reduction on metal electrodes (Cu, Fe and Co), in contrast to the NAD˙ radical pathway on carbon electrodes.

## Graphical abstract

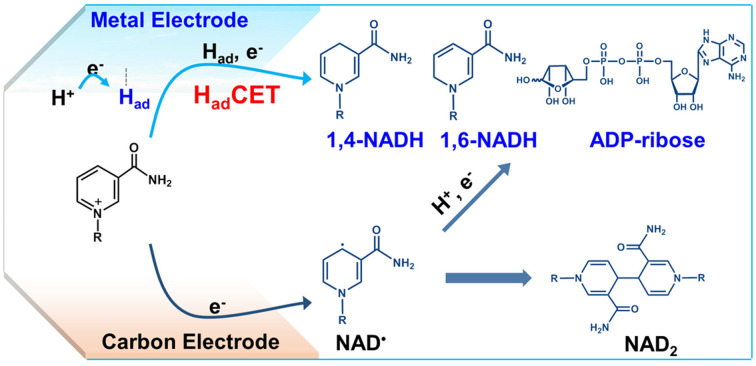

## Introduction

Most biocatalytic pathways depend on either of the two important cofactors, nicotinamide adenine dinucleotide (NAD^+^) or nicotinamide adenine dinucleotide phosphate (NADP^+^), which exist as redox couples with their corresponding reduced forms, NADH and NADPH (collectively abbreviated hereafter as NAD(P)H). NADH and NADPH differ in a phosphate group and function in different biological reactions in organisms. In natural photosynthesis, solar energy is harvested by photosystems, and the electrons and protons from the water oxidation reaction are utilized for NADPH production, which is then used as reducing equivalent for CO_2_ fixation dark reactions in the Calvin cycle.^1–3^ Nicotinamide adenine dinucleotide cofactor is a critical charge transfer mediator and energy carrier in photosynthesis and is involved in biocatalysis by oxidoreductases. NAD(P)H turns into its oxidized form (NAD(P)^+^) after donating two electrons and one proton and is difficult to use stoichiometrically for practical large-scale biocatalytic reactions.^4^ Therefore, efficient reduction of NAD^+^ to NADH with artificial catalysts is of significance for biocatalysis, understanding natural photosynthesis and constructing artificial photosynthetic systems for solar energy utilization.^5–14^

Previously, NAD^+^ has been reduced to NADH through biocatalysis with sacrificial reagents, such as formic acid,^15–18^ and photo(electro)catalysis with noble metal complexes such as [Cp*Rh(bpy)(H_2_O)]Cl_2_,^19–25^ which is coupled with enzyme-catalyzed reduction reactions.^19,24,26–31^ However, there are product separation difficulties and serious problems of mutual inactivation of molecular and enzymatic catalysts due to their interaction.^32–34^ Heterogeneous NAD^+^ hydrogenation to NADH with H_2_ as the reducing agent can avoid the above issues, but the selectivity for 1,4-NADH is relatively low.^35^ Electrocatalysis by metal catalysts with H_2_O providing protons and electrons for NAD^+^ reduction is another important way for artificial NADH production,^36–40^ but the formation of 1,6-NADH and NAD_2_ side products leads to a low selectivity for 1,4-NADH.^35–38,40–42^ The intrinsic reasons behind this issue are not well understood. It has been reported that NADPH production in natural photosynthesis proceeds through direct hydride transfer from the flavin adenine dinucleotide hydride (FADH^−^) to the nicotinamide controlled by ferredoxin–NADP^+^ reductase (FNR).^43^ For NAD^+^ reduction to NADH by artificial catalysts, it has been found that the transfer of two electrons results in the formation of 1,4-NADH and its 1,6-isomer, whereas the uptake of one electron leads to the formation of the NAD˙ radical that undergoes irreversible dimerization. Nevertheless, there is an ongoing debate on the mechanism of NAD^+^ reduction that the reaction may proceed through a direct electron transfer pathway with the NAD˙ radical as an intermediate or a hydride transfer mechanism.^21,23^ In addition, product analysis which has long been a thorny issue was only based on the grounds of preliminary evidence with carbon, Hg, and several metal catalysts without detailed product quantification and mechanism analysis. Therefore, it is of importance to study the mechanisms of the electrocatalytic NAD^+^ reduction reaction with different catalysts and to understand their differences from the pathway of selective 1,4-NAD(P)H production in nature, which is of significance for the exploration of more efficient artificial catalysts for NAD^+^ reduction to 1,4-NADH.

Herein, taking various metal and carbon materials as catalysts, the electrocatalytic NAD^+^ reduction reaction and the mechanisms are studied with systematic quantification of products. We find that the selectivity of 1,4-NADH is relatively high on Cu, Fe, and Co electrodes without forming NAD_2_ side products, different from the literature.^17^ In contrast, obvious formation of NAD_2_ is observed on the carbon electrode with low activity and selectivity for 1,4-NADH. And ADP-ribose is confirmed to be a fragmentation side product. With the reaction kinetics, H/D isotope effect, and electron paramagnetic resonance (EPR) analysis, we propose a hydrogen atom-coupled electron transfer (H_ad_CET) mechanism for the NAD^+^ reduction reaction on these metal electrodes in contrast to the NAD˙ radical process on the carbon electrode.

## Results and discussion

**Fig. 1:**
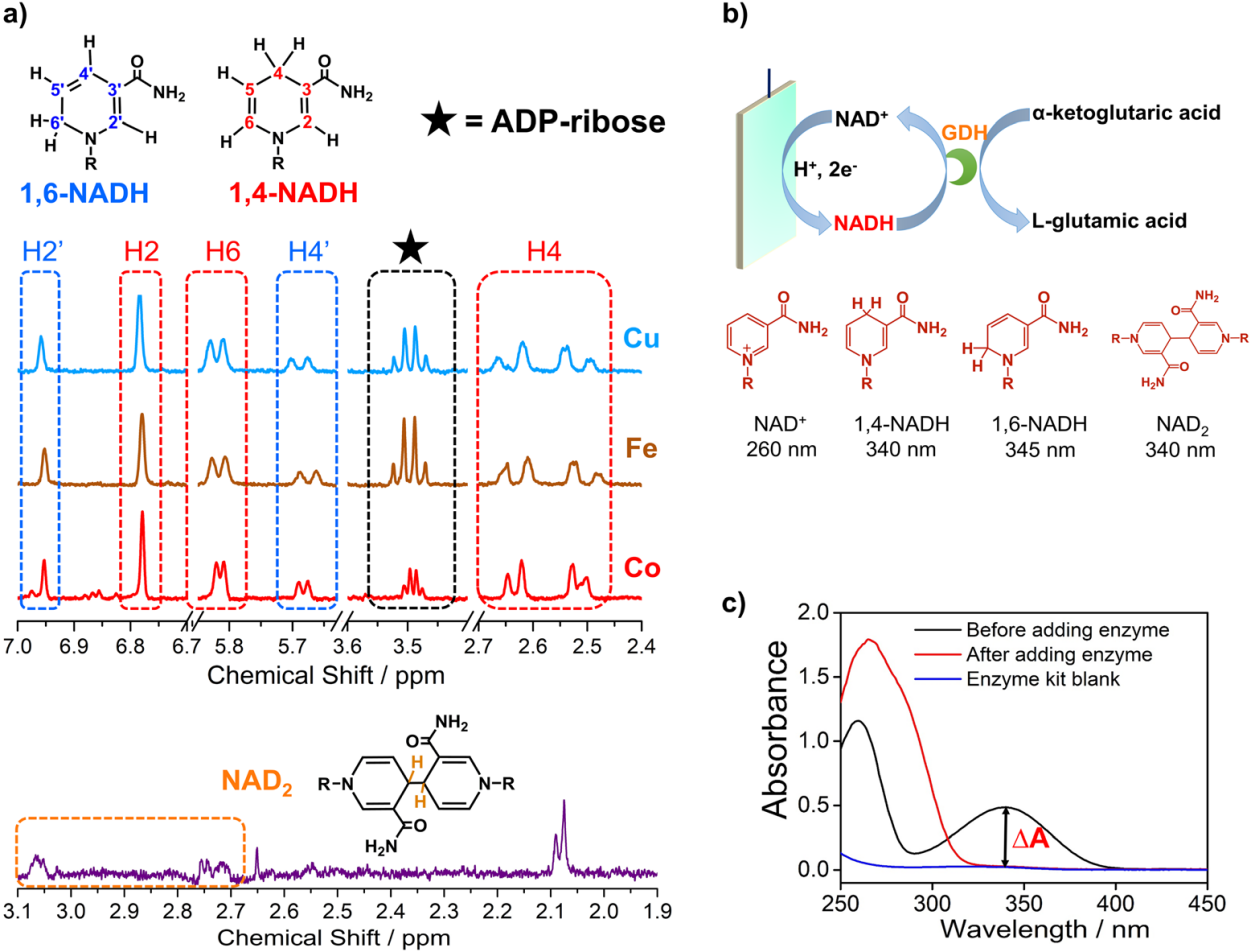
(a) The signals of 1,4-NADH (H2, H4, and H6), 1,6-NADH (H2′ and H4′), ADP-ribose, and NAD_2_ in the ^1^HNMR spectra of the products after electrocatalytic NAD^+^ reduction on Cu foam, Co foam, Fe foam, and carbon felt. Herein, the results of Cu, Fe, and carbon are measured with 400 MHz ^1^HNMR, while the result of Co is obtained with 700 MHz ^1^HNMR. Comparison of 400 MHz and 700 MHz ^1^HNMR is shown in Fig. S3.† (b, c) Schematic description of electrocatalytic NAD^+^ reduction and the determination of different products.

Electrocatalytic NAD^+^ reduction to NADH was studied with Cu, Fe, Co, and carbon electrodes (see the ESI† for the preparation and morphology details, Fig. S1†). NAD^+^ is fully consumed after the reaction judging from the disappearance of the ^1^H nuclear magnetic resonance (^1^HNMR) signals of NAD^+^ (Fig. S2†). Typical signals of 1,4-NADH with diagnostic chemical shifts at 2.6 ppm, 5.8 ppm, and 6.8 ppm appear after the reaction, suggesting that 1,4-NADH is produced (Fig. 1a, S2 and S3†). The change in UV-visible absorption spectra as a function of time indicates the consumption of NAD^+^ and generation of NADH (Fig. S4†). NAD_2_ was commonly considered as an important side product on metal electrodes such as Au and Hg.^44–46^ However, in our results, only 1,4-NADH and 1,6-isomer are produced without the observation of NAD_2_ with Cu, Fe, and Co electrodes (Fig. 1a, S2 and S3†), indicating a distinct reaction mechanism. In sharp contrast, with carbon electrodes, there are NAD_2_ signals in ^1^HNMR spectra after the reaction, consistent with previous reports.^34^ It is noteworthy that there is some ADP-ribose, which may be caused by the fragmentation of NAD^+^ and has rarely been reported before for NAD^+^ reduction.

More importantly, systematic quantitative analysis of products that has always been a tough issue was conducted combining UV-visible absorption spectra and NMR measurements (see the ESI† for details about the quantification of all products). The typical light absorption band of NAD^+^, 1,4-NADH, 1,6-NADH, and NAD_2_ is located at 260 nm, 340 nm, 345 nm, and 340 nm, respectively.^47^ Glutamate dehydrogenase (GDH) was used as an enzymatic catalyst for 1,4-NADH determination. The amount and selectivity of 1,4-NADH can be calculated from the change of absorbance (Δ*A*) before and after the enzyme catalyzed reaction, as shown in Fig. 1b and c. For the case of metal electrodes, the amount of 1,6-NADH is quantified from the residual absorbance after adding GDH. Then, the amount of ADP-ribose can be obtained from the difference of the initial NAD^+^ amount and NADH produced. And NAD_2_ can be quantified from the absorbance and the amount of NADH according to their molar extinction coefficient.

**Fig. 2:**
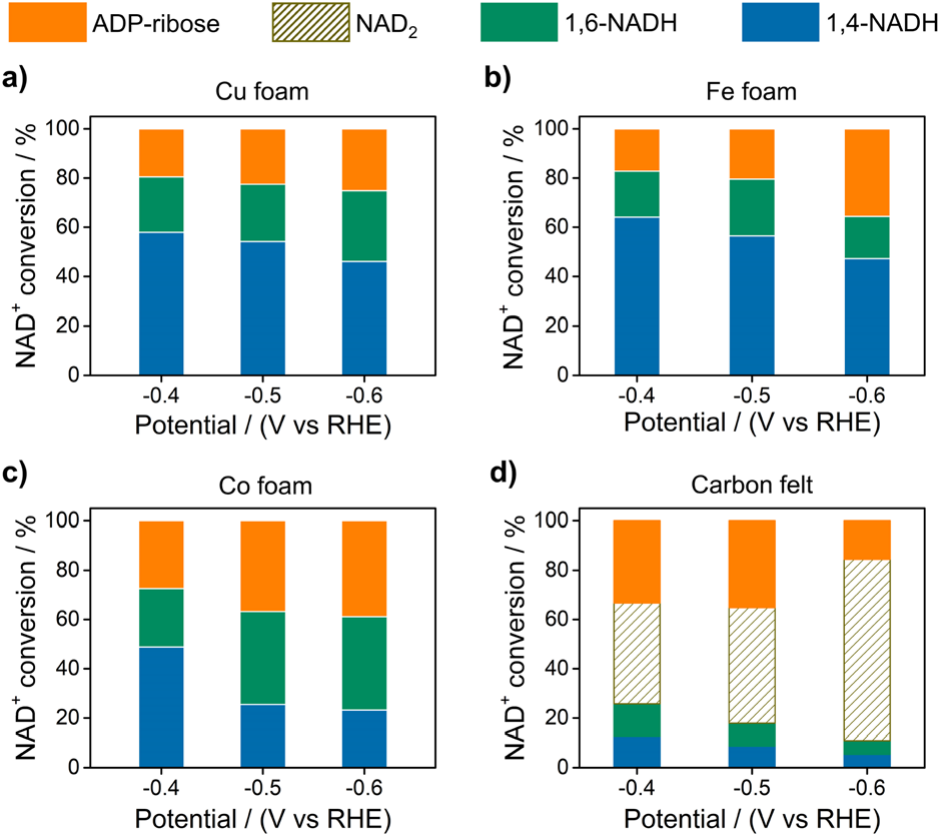
The conversion of NAD^+^ to different products on (a) Cu, (b) Fe, (c) Co foams, and (d) carbon felt at different potentials. Electrolyte: 0.1 M phosphate buffer (pH 7); catholyte: 12 mL electrolyte with an initial NAD^+^ concentration of 1 mM.

Fig. 2 displays the NAD^+^ conversion to different products when NAD^+^ is totally consumed. The highest yield of enzymatically active 1,4-NADH with Cu, Fe, and Co electrodes is about 57.9%, 64.1%, and 48.81% at −0.4 V *vs.* RHE (denoted as V_RHE_), respectively. In sharp contrast, the regeneration of 1,4-NADH is only 7.9% at −0.4 V_RHE_ on the carbon electrode with a high percentage of NAD_2_ (above 40%).

**Fig. 3:**
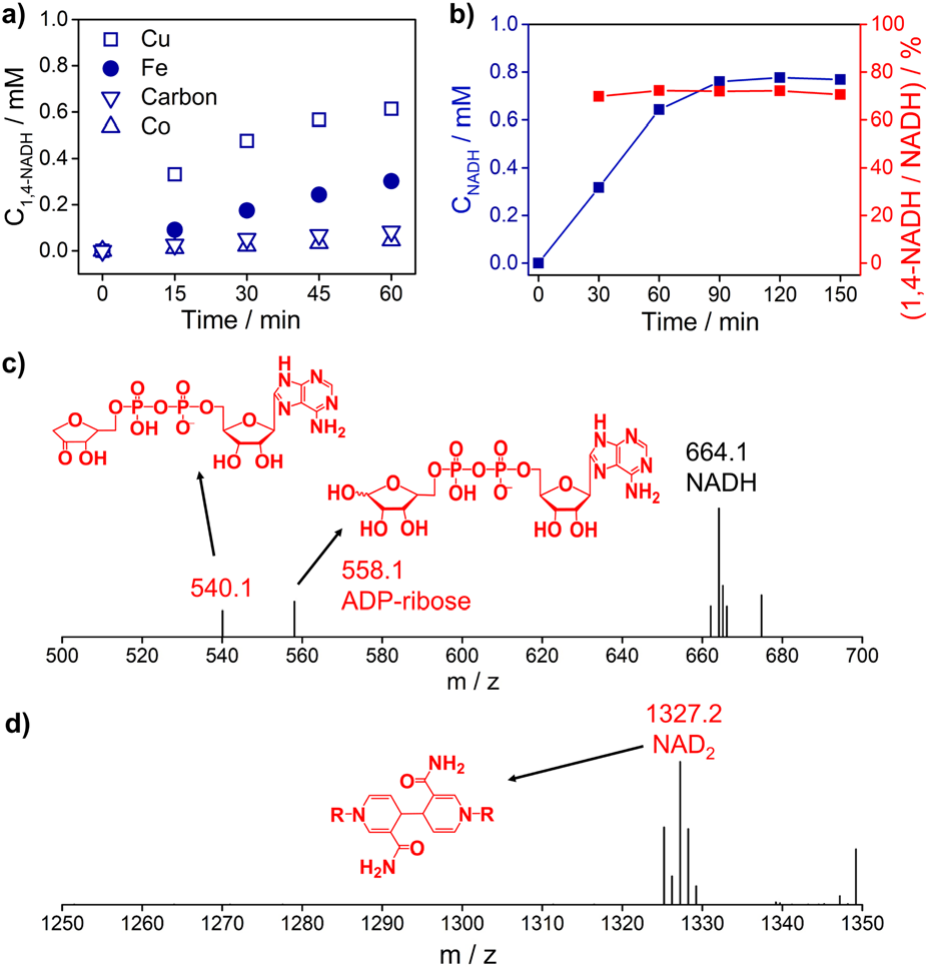
(a) The 1,4-NADH concentration trends on Cu foam, Fe foam, Co foam, and carbon felt under −0.5 V_RHE_. (b) The concentration of NADH and selectivity of 1,4-NADH *versus* total NADH (1,4-NADH + 1,6-NADH) concentration as a function of time. The electrospray ionization mass spectrometry (ESI-MS) of electrocatalytic NAD^+^ reduction products on (c) Cu foam and (d) carbon felt. Electrolyte: 0.1 M phosphate buffer (pH 7); catholyte: 12 mL electrolyte with an initial NAD^+^ concentration of 1 mM. ESI-MS full spectra of electrocatalytic NAD^+^ reduction products on Cu foam and carbon felt are shown in Fig. S6.†

Fig. 3a and S5† show the 1,4-NADH concentration trends during the NAD^+^ reduction reaction on the Cu, Fe, Co, and carbon electrodes at fixed potentials. The 1,4-NADH formation rate on the Cu electrode is highest among all of the tested electrodes. And the 1,4-NADH formation rate increases with the applied potential from −0.4 V_RHE_ to −0.5 V_RHE_ and then decreases at −0.6 V_RHE_ (Fig. S5†). As shown in Fig. 3a and b, the concentration of the aimed 1,4-NADH product and the total concentration of NADH (1,4-NADH + 1,6-NADH) increase with the reaction time, and the production rate decreases with the consumption of NAD^+^. However, the percentage of 1,4-NADH in NADH remains at around 71% with negligible decline as a function of time, that's to say the selectivity for 1,4-NADH does not change, indicating that the reaction mechanism is independent of the reaction degree. More interestingly, ADP-ribose is observed with both kinds of materials. To confirm the existence of ADP-ribose derivatives and NAD_2_ products, the electrolyte after the reaction was analyzed with electrospray ionization mass spectrometry (ESI-MS). Typical signals of ADP-ribose derivatives (*m*/*z* 558.1 and 540.1) and NAD_2_ (*m*/*z* 1327.2) can be detected, as shown in Fig. 3c, d and S6.†

**Fig. 4:**
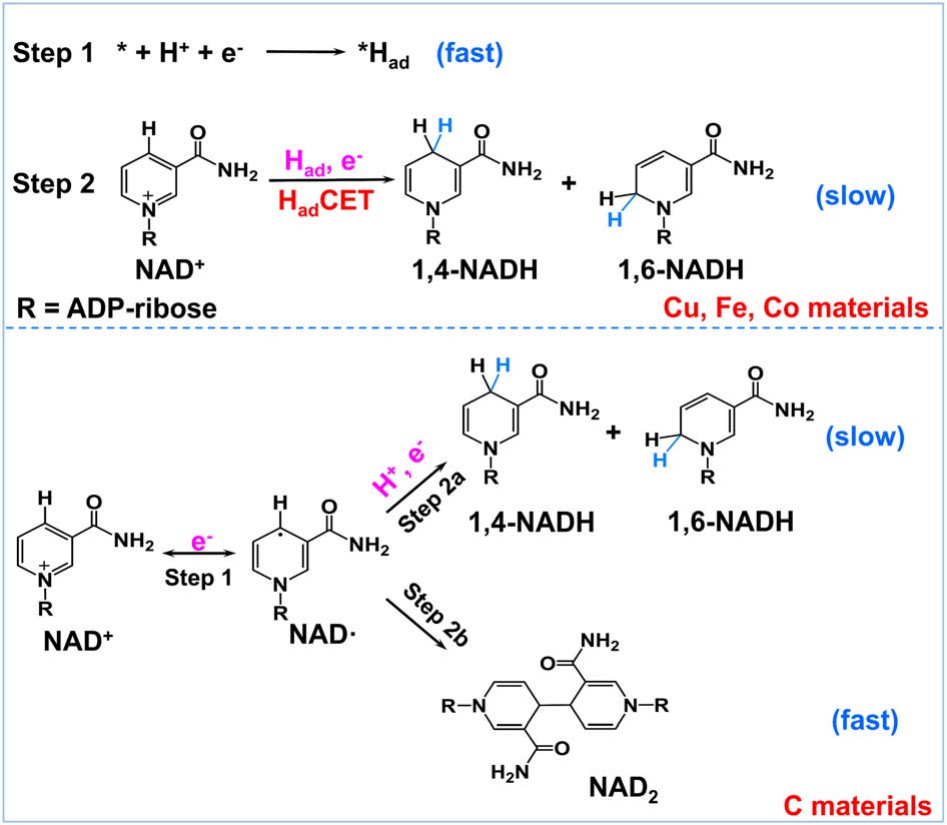
Schematic description of the possible reaction mechanism of NAD^+^ reduction on H_ad_-rich metal electrodes and H_ad_-poor carbon electrodes.

Encouraged by the above results, we propose a possible reaction mechanism for NAD^+^ reduction, as illustrated in Fig. 4. Considering that the nicotinamide moiety of NAD^+^ is positively charged, the H^+^ transfer as the initial step is difficult. For NAD^+^ reduction with organometallic homogenous catalysts, such as Rh complexes, it is well acknowledged that Rh–H is first formed, and then, NADH is produced through one-step hydride transfer between the reduced Rh–H and NAD^+^ by forming a special ring transition state.^21^ In previous reports, for (photo)electrocatalysis without a charge transfer mediator, the reaction mechanism was inconsistent. A hydride transfer mechanism has been proposed for NADH regeneration with the Pt/p-GaAs photocathode.^39^ However other studies performed on a variety of metal electrodes such as Au and Hg have led to a commonly established reduction mechanism that the first electron transfer is the initial step forming the NAD˙ radical, which tends to dimerize quickly and may also accept another electron and one proton forming 1,4-NADH and side products such as 1,6-NADH.^48^ Yet for the case of our tested metal catalysts, based on the above product analysis results, no NAD_2_ is produced in the reaction. Such being the case, we propose that no NAD˙ is produced in the reaction because the rate of NAD˙ dimerization is very fast with a rate constant of 3 × 10^6^ M s^−1^,^46^ much faster than forming NADH by accepting a proton and one electron.^48^ This means that the NAD^+^ reduction mechanism on the tested metal electrodes is quite different from the NAD˙ pathway.

In natural photosynthesis, NADPH is produced through the hydride transfer from FADH^−^ to NADP^+^ controlled by the FNR enzyme.^43^ On metal electrodes, hydride species are usually difficult to form and the hydride transfer mechanism can be excluded. Nevertheless, many organic, organometallic, and biological systems involving (C, N, O, S)–H and metal–H bonds, such as Ru-based water oxidation complexes, artificial and natural water oxidation clusters, hydrogenases, flavin cofactors and heme proteins, are related to the proton-coupled electron transfer (PCET) process.^49–51^ Natural organisms are masterful in managing PCET to drive the photosynthesis and nitrogen fixation reactions. It is worth noting that the surface of metal electrodes may be covered with many surface-adsorbed hydrogen atoms (H_ad_) as they have high proton activation ability, considering that they are widely used for hydrogen evolution or hydrogenation reactions. With these points in mind, we deduce that H_ad_ is primarily involved in the NAD^+^ reduction reaction on H_ad_-rich metal catalysts. To be specific, H_ad_ atoms are first formed *via* the Volmer process (* + H^+^ + e^−^ → *H_ad_, * is the active site). Second, NADH is produced *via* the reaction of H_ad_ and NAD^+^ coupled with an electron transfer (NAD^+^ + *H_ad_ + e^−^ → * + NADH). This H_ad_-coupled electron transfer (H_ad_CET) mechanism is similar and coincides with the commonly observed PCET process in natural biology except that the H_ad_ atom on the electrode surface is the hydrogen source for H_ad_CET here. It may occur *via* a stepwise electron–H_ad_ or H_ad_–electron transfer process, or through a concerted H_ad_–electron transfer process, which needs more research. Interestingly, we also evaluated the performance of Pt, Ni, Ru, and Ag for comparison and found that no or trace NADH is produced with these electrodes (Fig. S7†). It is inferred that the H_ad_ atoms formed on Pt, Ni, and Ru surfaces tend to form H_2_ molecules and the formation of H_ad_ is relatively difficult on the Ag surface, judging from their Δ*G*_H_ and exchange current density for hydrogen evolution in the literature (Table S1†).^52–54^ This means that a high coverage of H_ad_ on metal electrodes is favorable for NADH formation. In the case of the carbon electrode, it has poor proton activation ability and its surface is likely to have a low coverage of H_ad_. Therefore, the NAD^+^ reduction may follow the direct one-electron transfer and NAD˙ radical mechanism mentioned in the literature.^55^

**Fig. 5:**
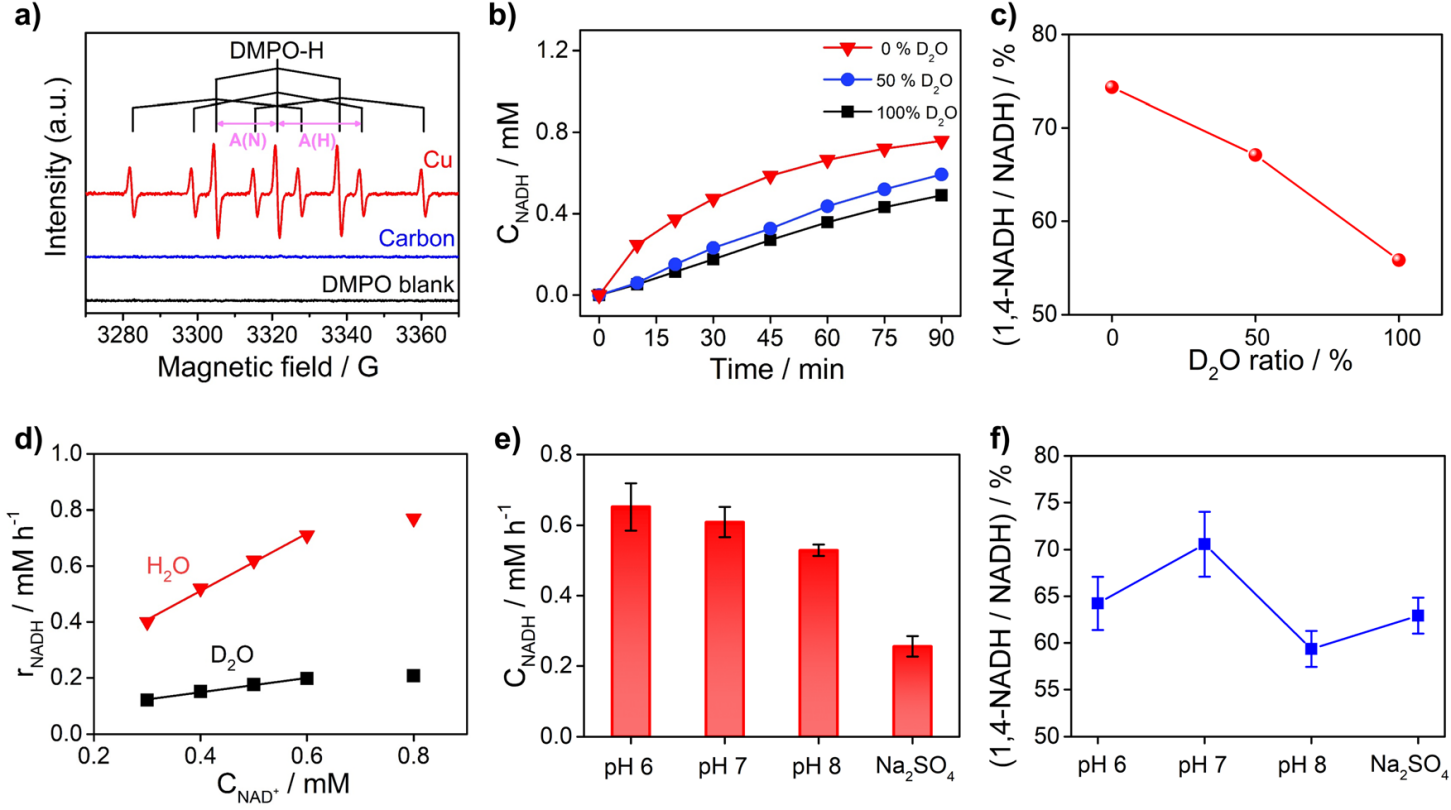
(a) EPR spectra in the X-band of the solutions obtained after electrolysis with Cu foam and carbon felt in 0.1 M phosphate buffer (pH 7) under argon using DMPO as the H˙ trapping reagent. The blank experiment is the EPR result of electrolyte without electrolysis after adding DMPO. (b) Activity and (c) selectivity of the NAD^+^ reduction reaction on Cu foam in 0.1 M phosphate buffer with different contents of D_2_O solvent. (d) Kinetic isotope effect of the NAD^+^ reduction reaction on Cu foam. (e) The activity and (f) selectivity of NADH regeneration at different pH and electrolytes. Electrolyte: 0.1 M phosphate buffer (pH 6, 7 and 8) and unbuffered 0.1 M Na_2_SO_4_ aqueous solution; catholyte: 12 mL electrolyte with an initial NAD^+^ concentration of 1 mM.

To obtain further insights into the reaction mechanism of NAD^+^ reduction, we used the EPR technique to detect the hydrogen species on the electrode surface. The H_ad_ atoms may diffuse into the nearby electrolyte forming H˙ radicals, which can be captured by the radical trapping reagent 5,5-dimethyl-1-pyrroline-*N*-oxide (DMPO, Fig. S8†). Fig. 5a displays the EPR results when adding DMPO to the NAD^+^-free electrolyte after electrolysis with Cu and carbon as working electrodes at −0.4 V_RHE_. The signals with Cu show an intensity ratio of 1 : 1 : 2 : 1 : 2 : 1 : 2 : 1 : 1 and their hyperfine coupling constants are *A*(N) = 16.43 G and *A*(H) = 22.68 G, which are consistent with the characteristic signals of DMPO–H.^56^ The DMPO–H signals can be obviously observed with Cu, while the EPR signals are negligible with the carbon electrode, which confirms that the surface of Cu is rich in H_ad_ in sharp contrast to carbon which is poor in H_ad_. And obvious DMPO–D signals can be observed in the EPR spectrum (Fig. S9†) of the solution after electrolysis with the Cu electrode in deuterated buffer, which consolidates our conclusion that there are many H_ad_ atoms on the Cu electrode surface. In addition, the probability of NADH production *via* the reaction between H˙ and NAD^+^ with the participation of another electron from the electrode is low. Therefore, the EPR results further rationalize that NADH on metal electrodes such as Cu, Fe, and Co is produced *via* the H_ad_CET process proposed above.

Furthermore, the critical role of H_ad_ for the NAD^+^ reduction reaction on metal electrodes was evidenced by the H/D isotope effect and reaction kinetic analysis. As shown in Fig. 5b, the activity of NADH production in H_2_O electrolyte is 2–4 times of that in electrolyte with D_2_O as the solvent, suggesting that hydrogen species play a key role in the rate-determining step (RDS) of NAD^+^ reduction to NADH. More interestingly, the selectivity for 1,4-NADH declines obviously with the increase of the D_2_O ratio in the electrolyte (Fig. 5c), which can be explained by the different coverage of H_ad_ and D_ad_ atoms due to their different production and consumption rates. In addition, as shown in Fig. 5d, the NADH formation rate (*r*) increases linearly with the initial NAD^+^ concentration from 0.3 mM to 0.6 mM (Fig. S10 and S11†), indicating a first-order reaction kinetics (*r* = *kC*_NAD^+^_, *k* = 1.03 h^−1^). Then, we explored the kinetic isotope effect (KIE, KIE = *k*_H_/*k*_D_) of the NAD^+^ reduction reaction in phosphate buffer (pH 7) with D_2_O as the solvent. A KIE value of 3.96 is calculated from the reaction rate constants in H_2_O and D_2_O electrolytes, suggesting a primary isotope effect. If the C–H formation is the only RDS, KIE should be around 7. A lower KIE means there is not only one RDS. The solvent isotope effect which results from isotopic differences in the medium can be excluded because H_2_O cannot directly participate in the NAD^+^ reduction reaction. The NADH formation by electrocatalytic NAD^+^ reduction involves the breaking of metal–H_ad_ and the electron transfer process, as shown in Fig. S12.† We speculate that the rates of metal–H_ad_ breaking and C–H formation of NADH may be similar. The RDSs of NAD^+^ reduction to NADH involve the breakage of the metal–H_ad_ bond and formation of the C–H bond during the hydrogenation of the nicotinamide group (Fig. S12†). The above results of the H/D isotope effect and KIE analysis all point to the H_ad_CET mechanism for NADH production mentioned above.

Moreover, the pH effect on the activity of NAD^+^ reduction with the Cu electrode was also studied (Fig. 5e and f). The total NADH (1,4- and 1,6-NADH) production follows the trend of pH 6 > pH 7 > pH 8. That's because the formation of H_ad_ is difficult under alkaline conditions, and thus, the coverage of H_ad_ is low and the reaction between NAD^+^ and H_ad_ is also slow. Interestingly, the selectivity of 1,4-NADH at pH 6 is lower than that of pH 7, indicating that the relatively high H_ad_ coverage at pH 6 may result in more random attacking of H_ad_ on NAD^+^.^57^ In addition, 1,4-NADH tends to decompose through acid-catalyzed protonation of the nicotinamide ring.^58^ For the control experiment with unbuffered Na_2_SO_4_ electrolyte, the activity and selectivity are obviously lower than those of neutral buffered electrolyte. This can be explained that there is an increase in local pH during the NAD^+^ reduction reaction as it is a proton-consuming reaction. So, careful pH control with buffered electrolyte is important for the NAD^+^ reduction reaction, and neutral phosphate buffer (pH 7) electrolyte delivers the highest activity for 1,4-NADH production. Therefore, these results of KIE and pH effects on the NAD^+^ reduction reaction, similar to the features for the PCET process,^49^ further supports the proposed H_ad_CET mechanism. In addition to the distinct coverage of H_ad_ on metal and carbon electrodes, their surface structure and the adsorption of NAD^+^ may be different in orientation and conformation, which needs more studies.

**Fig. 6:**
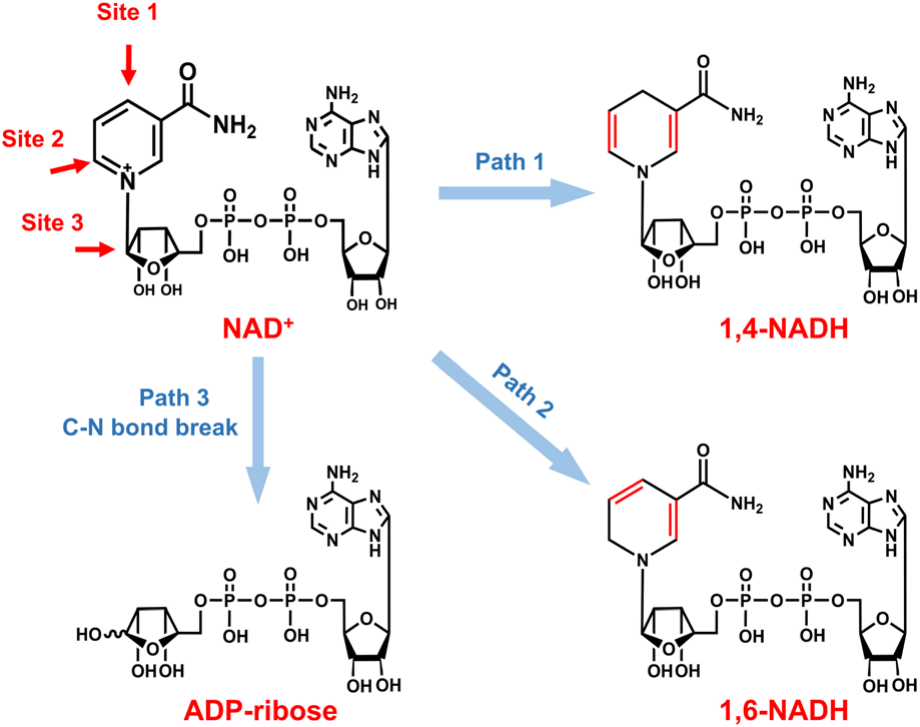
Possible reaction pathways of NAD^+^ with different attacking sites of H_ad_ and the corresponding products.

As for the formation of ADP-ribose, it was previously observed in the photocatalytic NADH oxidation reaction,^59^ but was rarely reported for the NAD^+^ reduction reaction. The possibility of the destructive hydrogenation reaction by H_2_ molecules can be excluded because there is no change of the NMR spectra after bubbling H_2_ through NAD^+^ solution for 3 h (Fig. S13†). Here, the formation of ADP-ribose may be caused by the fragmentation of NAD^+^ or the NAD˙ radical.^59,60^ For metal electrodes, there are many H_ad_ atoms on electrode surfaces. And three different sites in NAD^+^ may be attacked by H_ad_, as shown in Fig. 6. The hydrogenation of C4 (path 1) and C2 (path 2) sites leads to the formation of 1,4-NADH and 1,6-NADH. The positively charged N site in the nicotinamide group tends to suffer nucleophilic attack (path 3) by H_ad_, resulting in the breaking of the N–C bond and ADP-ribose fragments. For the carbon electrode, ADP-ribose comes from the fragmentation of the NAD˙ radical,^59^ and the amount of ADP-ribose is lower at more negative potential (Fig. 2d). However, the amount of ADP-ribose increases as the applied potential is more negative on metal electrodes (Fig. 2a–c), which shows little difference for Cu, Fe, and Co electrodes possibly as the adsorption configurations of NAD^+^ on these species are similar. Further work on suppressing ADP-ribose formation and promoting 1,4-NADH production by controlling the adsorption configuration of NAD^+^ and increasing the coverage of H_ad_ with rationally designed catalysts is still under research.

## Conclusions

Electrocatalytic NADH regeneration with various metal electrodes and carbon catalysts was investigated with quantification of all products, among which ADP-ribose is confirmed to be a side product caused by the fragmentation reaction. We find that the selectivity of bioactive 1,4-NADH is relatively high on Cu, Fe, and Co electrodes without forming side product NAD_2_, suggesting a mechanism involving H_ad_ on the surface rather than the previously reported NAD˙ intermediate. In contrast, there is obvious formation of NAD_2_ for the carbon electrode with low selectivity for 1,4-NADH. We propose that the formation of NADH on these metal electrodes proceeds *via* a H_ad_CET mechanism, in contrast to the direct electron transfer and NAD˙ radical pathway on carbon electrodes.

## Author contributions

Fengyuan Liu and Chunmei Ding conceived the project and wrote the manuscript under the supervision of C. Li. All authors discussed the results and contributed to manuscript editing.

## Conflicts of interest

The authors declare no competing financial interest.

## Supplementary Material

SC-013-D2SC02691K-s001
