# Supplementary material for: Electrocatalytic NAD+ reduction via hydrogen atom-coupled electron transfer
Source: Chem Sci. 2022 Oct 24;13(45):13361–7. doi: 10.1039/d2sc02691k (PMC9682901; doi:10.1039/d2sc02691k)
Supplement: SC-013-D2SC02691K-s001 [file SC-013-D2SC02691K-s001.pdf]

Electronic Supplementary Material (ESI) for Chemical Science.
This journal is © The Royal Society of Chemistry 2022

Electronic Supplementary Information:

Electrocatalytic NAD^+ Reduction via Hydrogen-Atom-Coupled Electron Transfer

Fengyuan Liu, [‡ab] Chunmei Ding, [‡bc] Shujie Tian,[bc] Sheng-Mei Lu,[bc] Chengcheng Feng,[bd] Dandan Tu,[bc] Yan Liu,[bc] Wangyin Wang,[bc] Can Li*[abc]

[a] Zhang Dayu School of Chemistry, Dalian University of Technology, Dalian 116024, Liaoning, China

[b] State Key Laboratory of Catalysis, Dalian Institute of Chemical Physics, Chinese Academy of Sciences; Dalian National Laboratory for Clean Energy, Dalian 116023.

[c] University of Chinese Academy of Sciences, Beijing 100049, China.

[d] School of Chemistry and Materials Science, University of Science and Technology of China, Hefei 230026, China

‡ These authors contributed equally.

* Corresponding author: canli@dicp.ac.cn

Contents

1. Chemicals and materials S2

2. Preparation and pre-treatment of electrodes S2

3. Electrochemical measurements S3

4. Quantification of various NAD^+ reduction products S3

5. NMR measurements S5

6. EPR measurements S5

7. H/D isotope experiment S6

Fig. S1 SEM images S7

Fig. S2 ^{1}HNMR spectra of NAD^+, 1,4-NADH and the NAD^+ reduction products S8

Fig. S3 400 MHz ^{1}HNMR spectra of the products on Cu, Fe, Co foams and 700 MHz ^{1}HNMR spectrum of Co foam S9

Fig. S4 UV-visible spectra S10

Fig. S5 Activity of 1,4-NADH production with Cu, Fe, Co and carbon electrodes S11

Fig. S6 The full spectrum and close shots of ESI-MS NAD^+ reduction products S12

Fig. S7 Activity of 1,4-NADH production with Pt, Ni, Ru and Ag electrodes S13

Table S1 ΔG_H and exchange current density of various metals S14

Fig. S8 Chemical structure of DMPO and schematic description of NADH formation and DMPO-H detection S15
Fig. S9 EPR spectra of electrolysis with Cu electrode in phosphate buffer deuterium solution S16
Fig. S10 Activity of NAD^+ reduction on Cu in phosphate buffer aqueous solution S17
Fig. S11 Activity of NAD^+ reduction on Cu in phosphate buffer deuterium solution........ S18
Fig. S12 The schematic description of KIE of NAD^+ reduction reaction on Cu. S19
Fig. S13 The ^{1}HNMR spectra of NAD^+ after stirring under air, Ar and H_2 atmosphere S20
References S21

1. Chemicals and materials

All chemicals were analytical grade and were used as purchased without further purification. The details are as follows: Na_2HPO_4 (99%, Sinopharm, China), NaH_2PO_4 (99%, Sinopharm, China), β-Nicotinamide adenine dinucleotide (95%, Sigma-Aldrich), reduced β-Nicotinamide adenine dinucleotide (99%, Sigma-Aldrich), Glutamate dehydrogenase (Sigma-Aldrich, G2501), α-Ketoglutaric acid (98%, Aladdin), $(NH_4)_2SO_4$ (99%, Macklin), D_2O (CIL), H_2O (>18 MΩ•cm, produced by PURELAB Ultra system), Cu foam (Guangjiayuan, China), Fe foam (Hui'eteng, China), Co foam (Hui'eteng, China), Ni foam (Guangjiayuan, China), Ag (Alfa Aesar), carbon felt (Delijia, China), $RuCl_3$ (99.98%, Sigma-Aldrich), 5,5-dimethyl-1-pyrroline-N-oxide (Dojindo).

2. Preparation and pre-treatment of electrodes

Cu, Co, Fe, Ni foams were cleaned by ultrasonication in ethanol for 15 min, then sealed by 704 silicone sealant exposing an area of 1 cm^2. Before electrochemical measurements, they were cleaned by ultrasonication in 1 M HCl for 5 min to remove the native oxide layer and then rinsed with pure water and dried with Ar stream.

The Ru electrode was prepared by depositing Ru on the Ni foam substrate. A cleaned Ni foam was immersed in de-aerated 0.1 M HCl containing 0.01 M $RuCl_3$ for 3 h.

The Pt electrode was cleaned by ultrasonication in 3 M H_2SO_4 for 30 min before use and then rinsed with pure water and dried with Ar stream. Then, it was sealed by 704 silicone sealant exposing an area of 1 cm^2.

The Ag electrode was cleaned by ultrasonication in ethanol for 15 min, then sealed by 704 silicone sealant exposing an area of 1 cm^2. Before electrochemical measurements, it was cleaned by ultrasonication in 3 M H_2SO_4 and then rinsed with pure water and dried with Ar stream.

3. Electrochemical measurements

All electrochemical measurements were conducted in a three-electrode setup with Pt counter electrode (2 cm × 0.5 cm) and Ag/AgCl (with saturated KCl) reference electrode. All potentials were controlled by a potentiostat (CHI 660e) without iR compensation.

The NADH regeneration reactions were conducted by controlled-potential electrolysis in an H-type cell containing 0.1 M sodium phosphate electrolyte, and the catholyte is 12 mL electrolyte with an initial NAD^+ concentration of 1 mM with a Nafion-117 membrane. Electrolytes were pre-saturated and bubbled with Ar under magnetic stir during measurements. Cu foam and other electrodes were used as work electrodes for NAD^+ reduction at applied potentials either -1.01 V, -1.11 V or -1.21 V vs. Ag/AgCl.

4. Quantification of various NAD^+ reduction products

The concentration of NADH was quantitatively determined by UV-visible light absorption and ^{1}HNMR measurements. Firstly, NADH was produced in 12 mL 0.1 M phosphate buffer (pH 7) catholyte with an initial NAD^+ concentration of 1 mM. After electrolysis, the NADH concentration was determined by UV-visible light absorption. The typical light absorption bands of NAD^+, 1,4-NADH, 1,6-NADH and NAD_2 are located at 260 nm, 340 nm, 345 nm and 340 nm, respectively.[1]

And the molar absorption coefficients (ε) of 1,4-NADH, 1,6-NADH and NAD_2 are 6220 $M^{-1} cm^{-1}$, 6580 $M^{-1} cm^{-1}$, and 6650 $M^{-1} cm^{-1}$, respectively.[1-2] The thickness of the cuvette for UV-visible light absorption measurements is 1 cm. The solution was diluted by 25 times with 0.1 M phosphate buffer before measuring the UV-visible light absorption to guarantee the applicability of Lamber-Beer principle.

The initial NAD^+ reactant was all consumed judging from the disappearance of its ^{1}HNMR signal. To determine the amount of 1,4-NADH, 20 mg α-ketoglutaric acid and 130 mg $(NH_4)_2SO_4$ were added to 10 mL electrolyte, then 9 unit glutamate dehydrogenase (GDH) was added. After enzyme catalysis for 15 min under stirring, 0.1 mL solution was extracted and diluted by adding 2.4 mL H_2O for UV-visible absorption measurements on a UV-vis spectrophotometer (Gary-55). The absorbance was monitored until reaching a final constant value, where the entire active 1,4-NADH formed during the electrolysis was consumed by the enzymatic reaction. Thus, the concentration of enzymatically-active 1,4-NADH formed in electrolysis process can be calculated from the change of UV-visible light absorption (ΔA) before and after the enzymatic reaction through equation (1).

$$C_{1,4\text{-NADH}} = [A_0 - (A_t - A_e)] / \varepsilon_{1,4\text{-NADH}} \qquad (1)$$

where A_0 is the initial absorbance prior to enzymatic catalysis reaction, A_t is the final absorbance after enzymatic reaction and A_e is the absorbance of α-ketoglutaric acid, $(NH_4)_2SO_4$ and GDH.[3]

For the cases of metal electrodes, there was no NAD_2 product. So the amount of 1,6-NADH was quantitatively determined from absorbance after enzymatic reaction， A_t (equation (2)), and then ADP-ribose amount was obtained by the difference of the initial amount of NAD^+ (which was all converted after the reaction) and amount of NADH (1,4-NADH + 1,6-NADH) produced, as equation (3) shows.

$$C_{1,6\text{-NADH}} = (A_t - A_e) / \varepsilon_{1,6\text{-NADH}} \quad (2)$$

$$C_{\text{ADP-ribose}} = C_{\text{NAD}^+} - C_{1,4\text{-NADH}} - C_{1,6\text{-NADH}} \quad (3)$$

For the case of a carbon felt, the amount of 1,4-NADH was determined according to equation (1) as mentioned above. The amount of 1,6-NADH was determined from the ratio of 1,4-NADH and 1,6-NADH based on its ^{1}HNMR data according to equation (4). Then the light absorption of 1,6-NADH ($A_{1,6\text{-NADH}}$) was calculated according to equation (5). The light absorption of NAD_2 can be calculated by subtracting the $A_{1,6\text{-NADH}}$ from A_t, and then the concentration of NAD_2 can be obtained according to equation (6). And then ADP-ribose amount was obtained by the difference of the initial amount of NAD^+ and the total amount of other products, equation (7).

$$C_{1,6\text{-NADH}} = C_{1,4\text{-NADH}} \times \text{Ratio}_{1,6\text{-NADH} / 1,4\text{-NADH in NMR}} \quad (4)$$

$$A_{1,6\text{-NADH}} = C_{1,6\text{-NADH}} \times \varepsilon_{1,6\text{-NADH}} \quad (5)$$

$$C_{\text{NAD2}} = [A_t - A_{1,6\text{-NADH}}] / \varepsilon_{\text{NAD2}} = [A_t - C_{1,6\text{-NADH}} / \varepsilon_{1,6\text{-NADH}}] / \varepsilon_{\text{NAD2}} \quad (6)$$

$$C_{\text{ADP-ribose}} = C_{\text{NAD}^+} - C_{1,4\text{-NADH}} - C_{1,6\text{-NADH}} - 2 \times C_{\text{NAD2}} \quad (7)$$

5. NMR measurements

The NAD^+ reduction products of Cu, Fe, Co foams and carbon felt were analyzed by ^{1}HNMR (Bruker 400 MHz) by adding 0.2 mL D_2O into 0.6 mL electrolyte after reaction. The NAD^+ reduction products of Co foam were also analyzed by ^{1}HNMR (Bruker 700 MHz) by adding 0.2 mL D_2O into 0.6 mL electrolyte after reaction.

6. EPR measurements

The existence of hydrogen radical (H·) was verified by EPR measurements (Bruker A200). The X-band EPR was measured at 9.325GHz and room temperature. The electrolysis was conducted by controlled-potential electrolysis in an H-type cell containing 0.1 M sodium phosphate electrolyte,

and the catholyte is 6 mL electrolyte with a Nafion-117 membrane. Electrolytes were pre-saturated and bubbled with Ar under magnetic stir during measurements. Metal and carbon electrodes were used as work electrodes.

The electrocatalysis was run at -1.01 V vs. Ag/AgCl for 5 min to enable equilibrium between H· formation and quenching. Then 60 μL DMPO was injected to electrolyte near the working electrode. After 10 min electrolysis, 1 mL electrolyte was frozen in liquid nitrogen and stored under -20 °C before EPR measurement.

7. H/D isotope experiment

In H/D isotope experiment, the electrolysis was conducted by controlled-potential electrolysis in an H-type cell containing 0.1 M sodium phosphate H_2O or D_2O electrolyte, and the catholyte is 12 mL electrolyte with a Nafion-117 membrane. Electrolytes were pre-saturated and bubbled with Ar under magnetic stir during measurements. A Cu foam (1 cm^2) was used as the work electrode.

In activity and selectivity experiments, 100 μL electrolyte after the reaction was extracted from the cell and diluted to 2.5 mL for UV-vis determination every 15 min.

In KIE experiments, 100 μL electrolyte after the reaction electrolyte was extracted from the cell and diluted to 2.5 mL for UV-vis determination every 5 min.

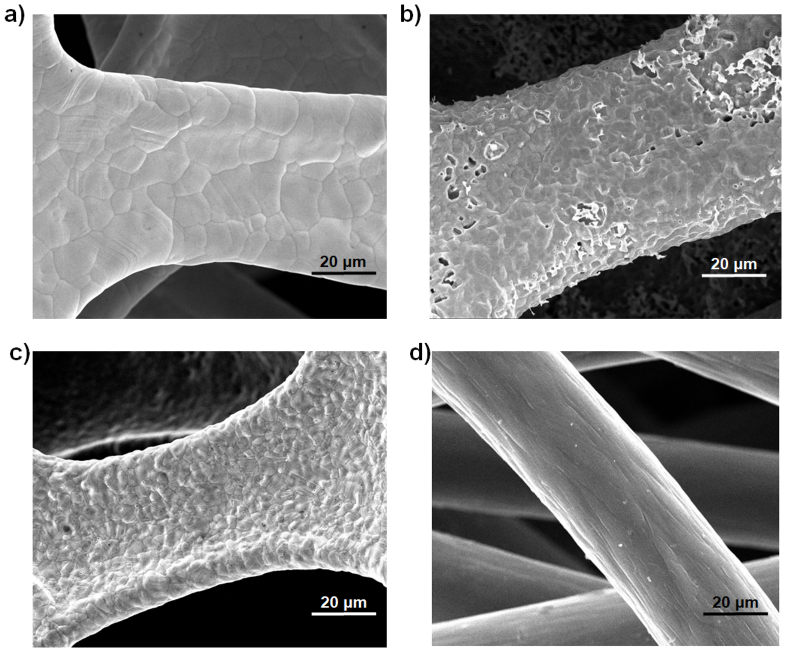

Fig. S1 SEM image of the (a) Cu foam, (b) Fe foam, (c) Co foam and (d) carbon felt used in this work.

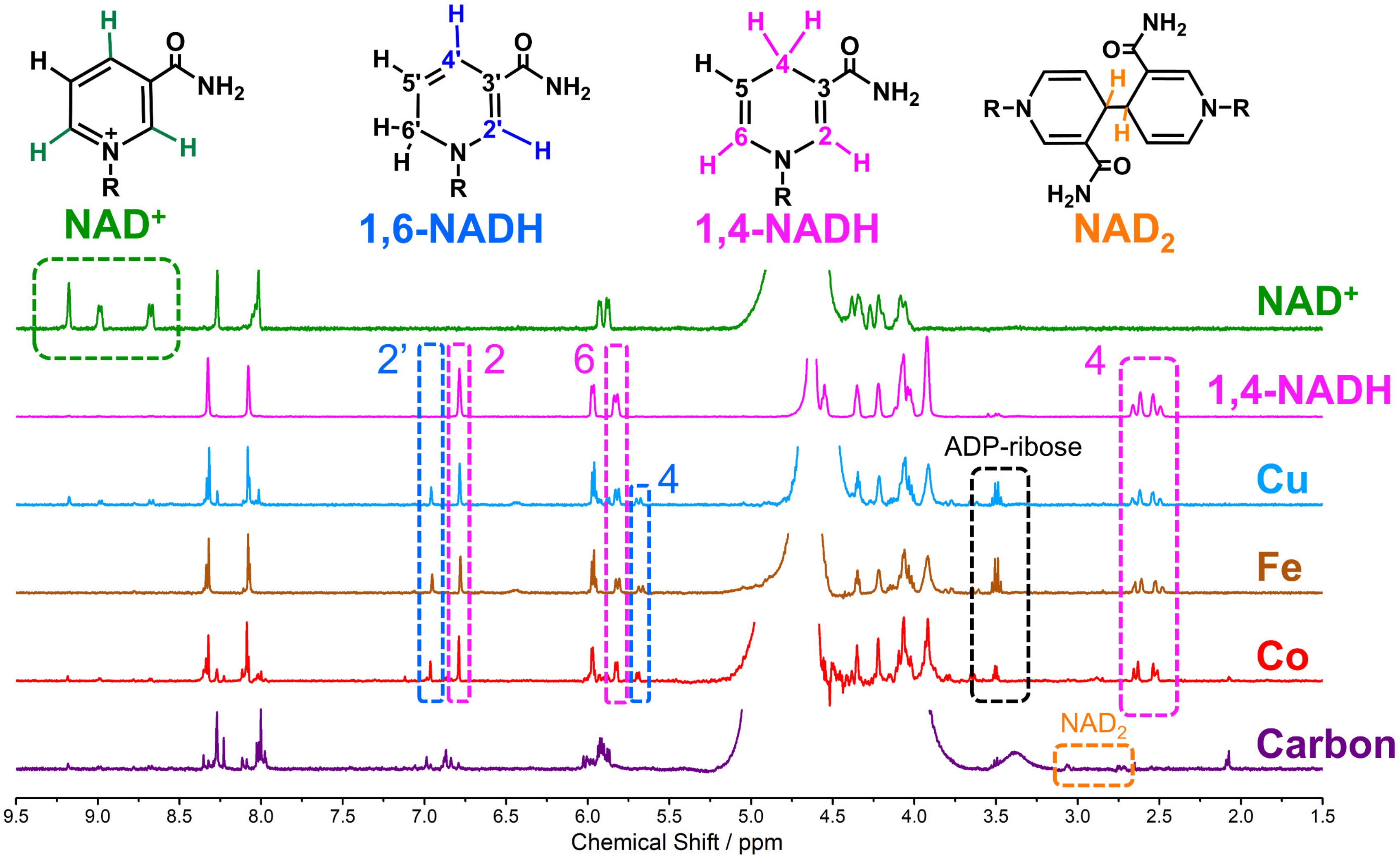

Fig. S2 The ^{1}HNMR spectra of commercial NAD^+, commercial 1,4-NADH and the products after electrocatalytic NAD^+ reduction on Cu, Fe, Co and carbon electrodes.

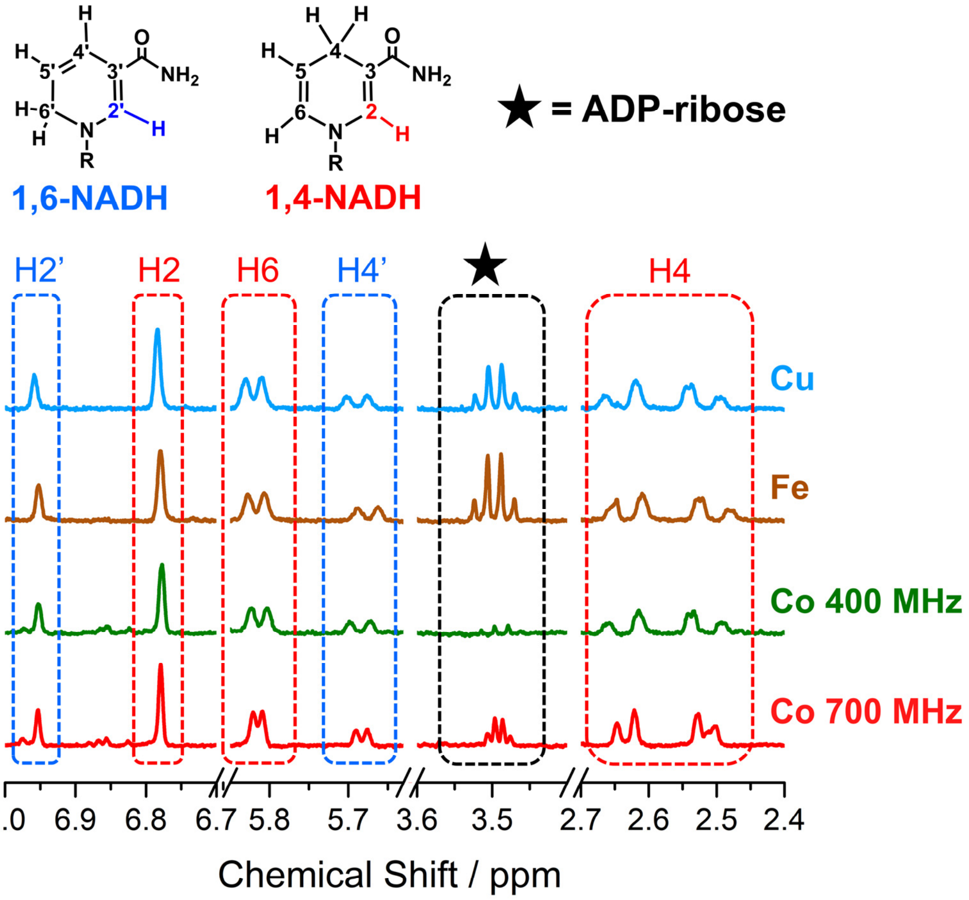

Fig. S3 The signals of 1,4-NADH (H2, H4, H6), 1,6-NADH (H2', H4'), ADP-ribose in the 400 MHz ^{1}HNMR spectra of the products on Cu foam, Fe foam, Co foam and 700 MHz ^{1}HNMR spectrum of the products on Co foam after electrocatalytic NAD^+ reduction.

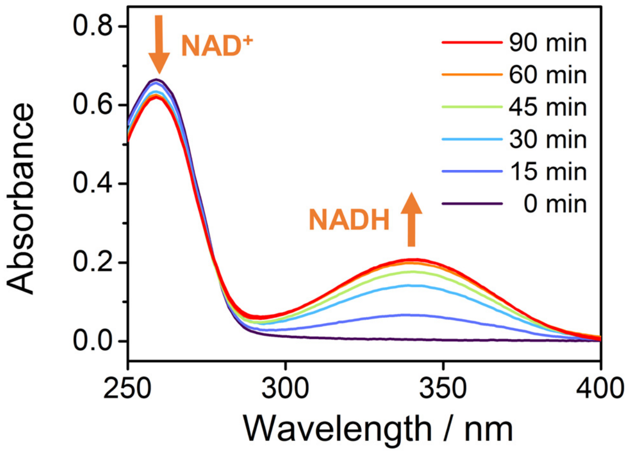

Fig. S4 Change of UV-visibe spectra profile as a function of time which indicates the consumption of NAD^+ and generaton of NADH on Cu foam at -0.4 V_{RHE}. Electrolyte: 0.1 M phosphate buffer (pH 7); Catholyte: 12 mL electrolyte with an initial NAD^+ concentration of 1 mM.

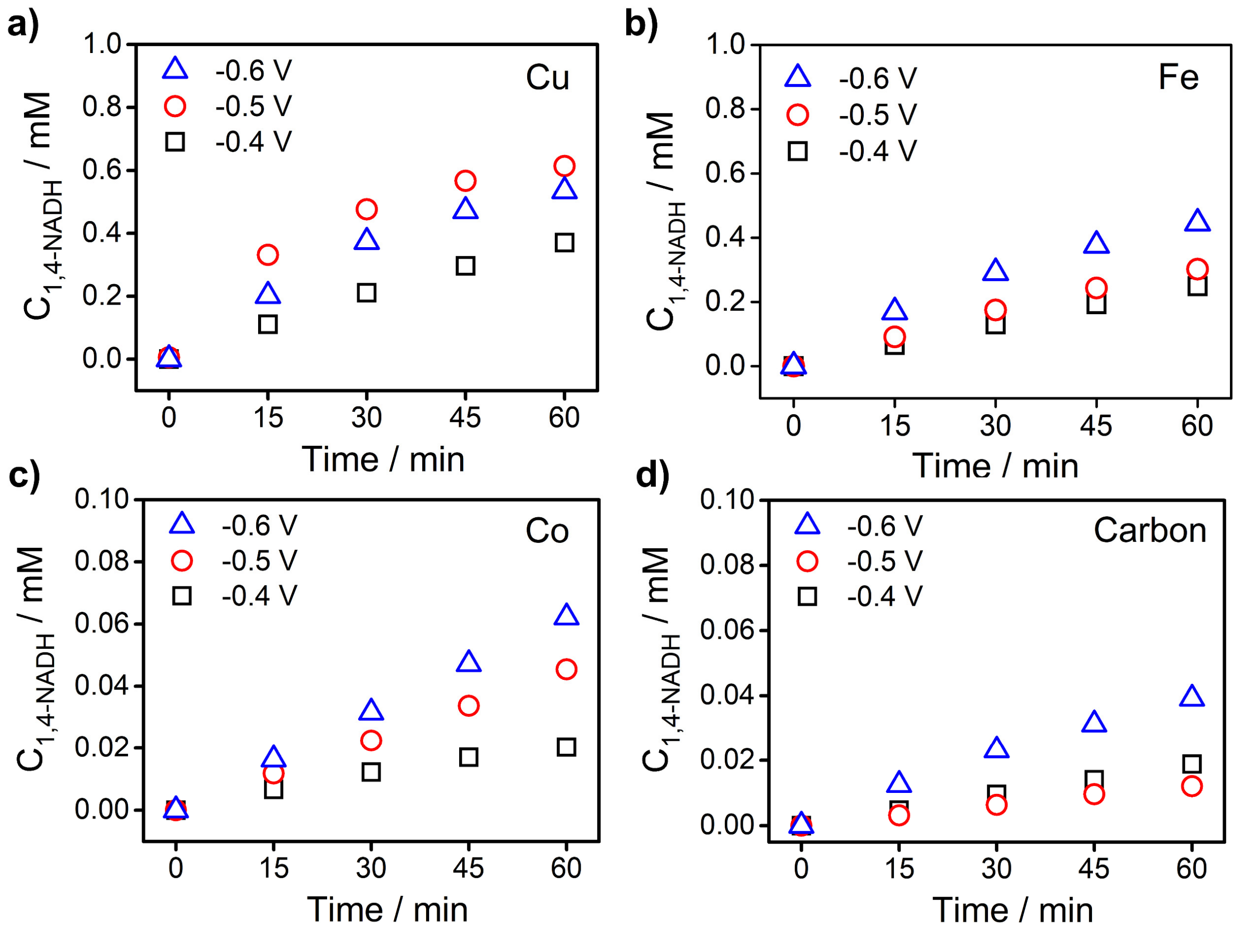

Fig. S5 The 1,4-NADH concentration trends on (a) Cu, (b) Fe, (c) Co and (d) carbon electrodes under different potentials. Electrolyte: 0.1 M phosphate buffer (pH 7); Catholyte: 12 mL electrolyte with an initial NAD^+ concentration of 1 mM.

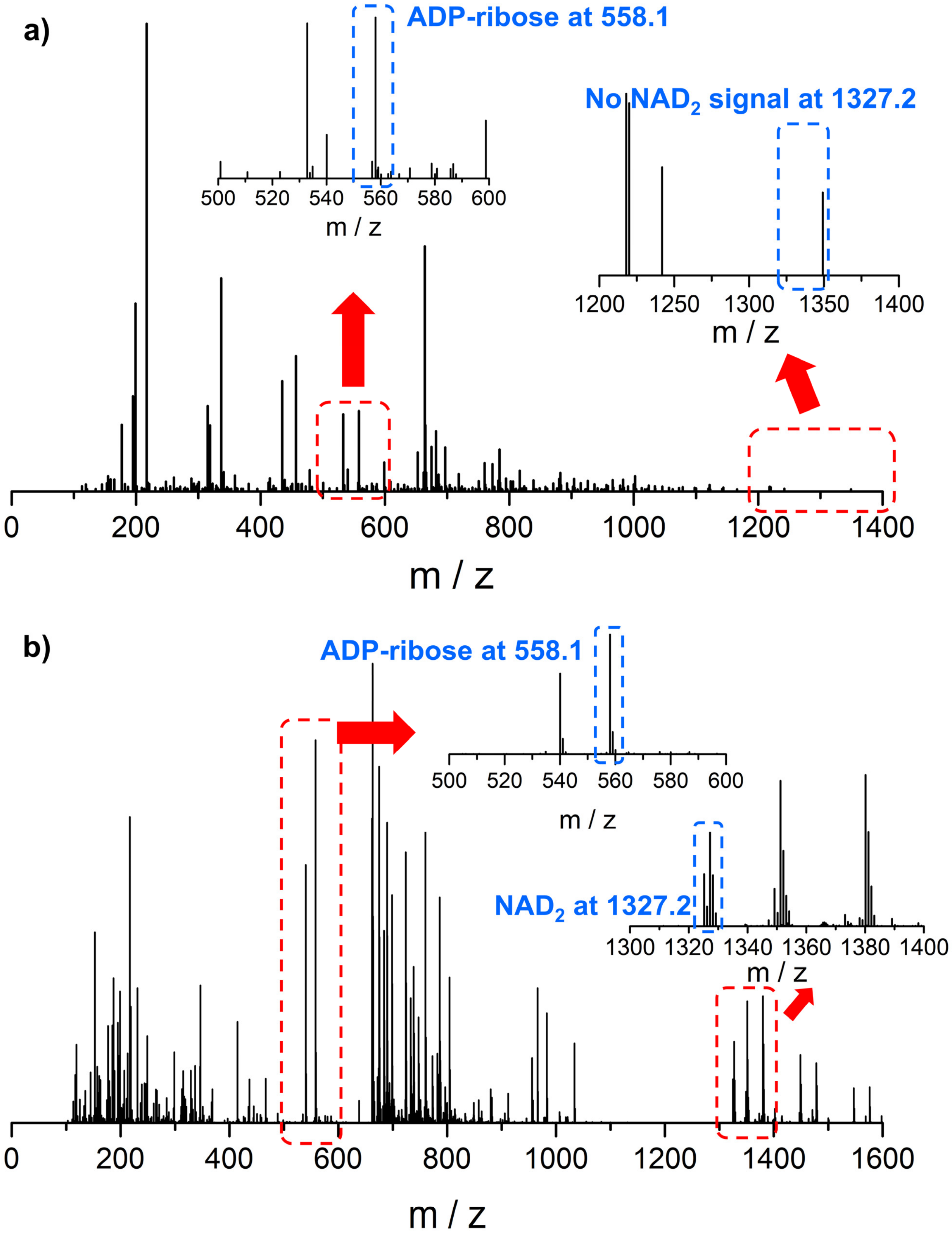

Fig. S6 The full spectrum and close shots of electrospray ionization mass spectrometry (ESI-MS) of electrocatalytic NAD^+ reduction products on (a) Cu foam and (b) carbon felt. Electrolyte: 0.1 M phosphate buffer (pH 7); Catholyte: 12 mL electrolyte with an initial NAD^+ concentration of 1 mM.

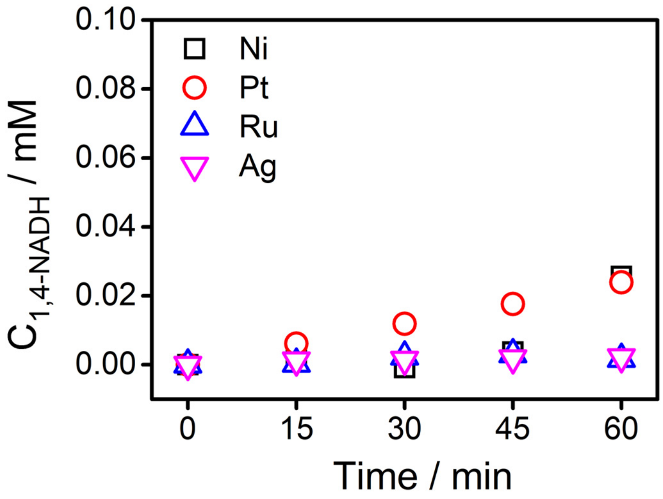

Fig. S7 The 1,4-NADH concentration trends on Ni, Pt, Ru/Ni and Ag electrodes under -0.4 V vs RHE. Electrolyte: 0.1 M phosphate buffer (pH 7); Catholyte: 12 mL electrolyte with an initial NAD^+ concentration of 1 mM.

Table S1 ΔG_H and exchange current density of various metals for hydrogen evolution adapted from the literatures.[4-6]

Metals	ΔG_H	$\log(j_0(mA/cm^{-2}))$
Pt	-0.066	-2.66
Ni	-0.13	-5.2
Ru	-0.30	—
Co	-0.16	-5.32
Fe	-0.37	—
Cu	0.28	-5.37
Ag	0.62	-7.8

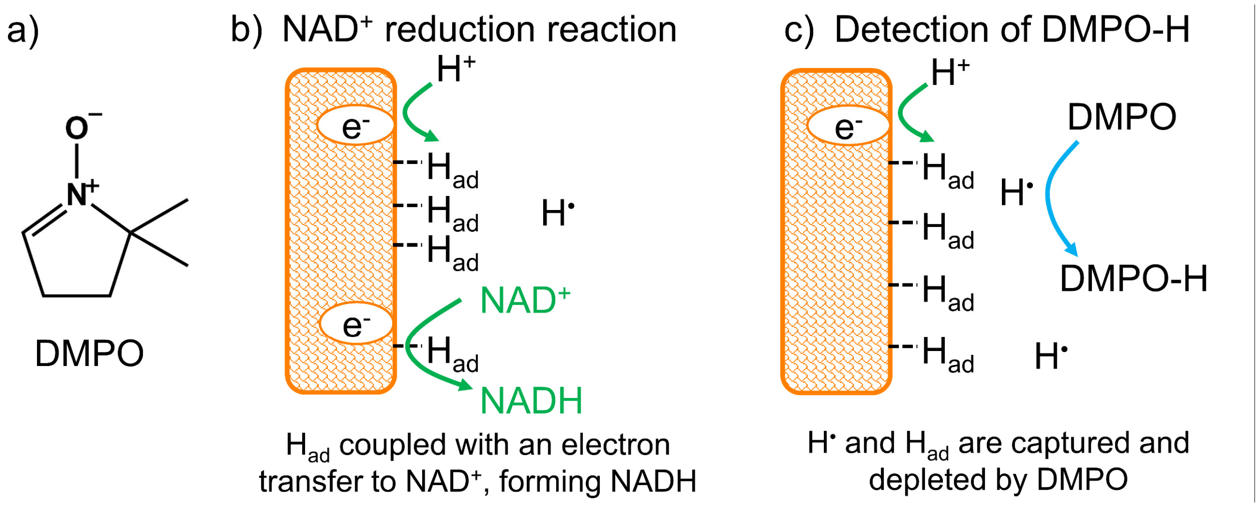

Fig. S8 (a) Chemical structure of DMPO (5,5-Dimethyle-1-pyrroline N-oxide). (b) The schematic description of the mechanism of NADH formation and (c) the detection of DMPO-H.

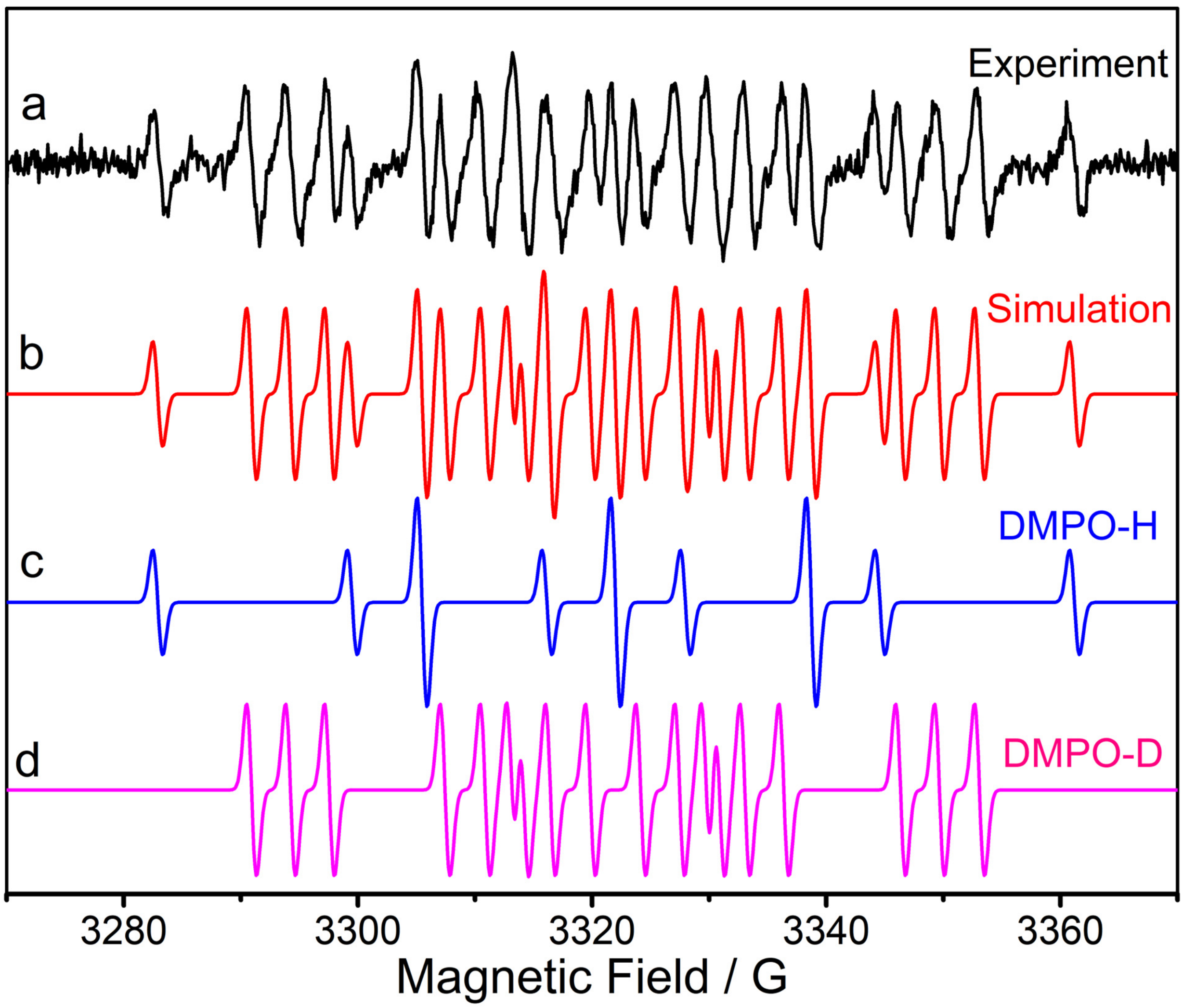

Fig. S9 (a) The experimental EPR spectrum by in X-band of the solutions obtained after electrolysis with Cu electrode in 0.1 M phosphate buffer with D_2O solvent under argon using DMPO as the radical trapping reagent. (b) The spectrum obtained by simulation and fitting by MATALAB. The EPR spectra of (c) DMPO-H and (d) DMPO-D extracted from the simulated spectrum.

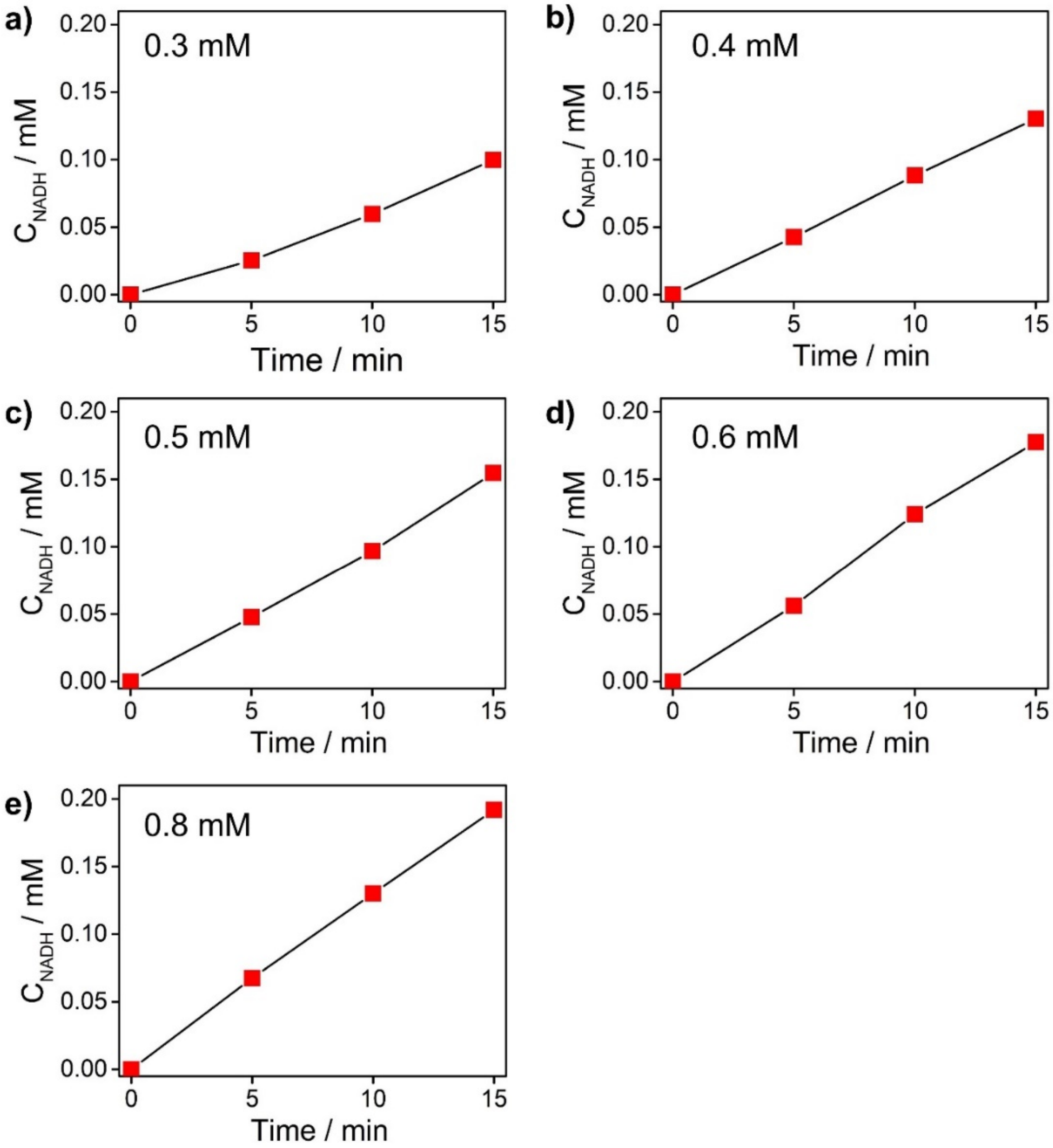

Fig. S10 The activity of NAD^+ reduction reaction in the first 15 min on Cu foams in phosphate buffer aqueous solution.

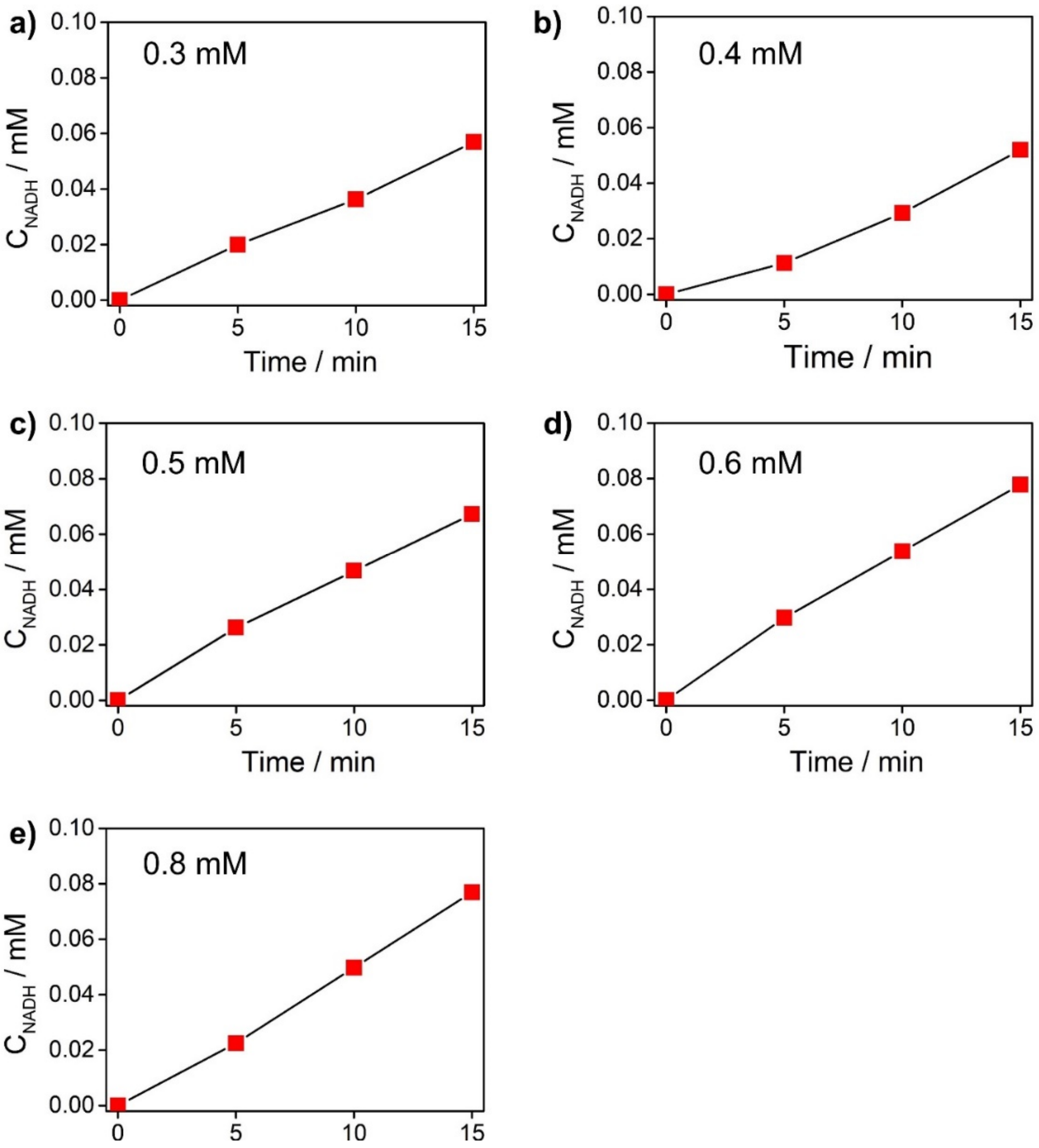

Fig. S11 The activity of NAD^+ reduction reaction in the first 15 min on Cu foams in phosphate buffer deuterium aqueous solution.

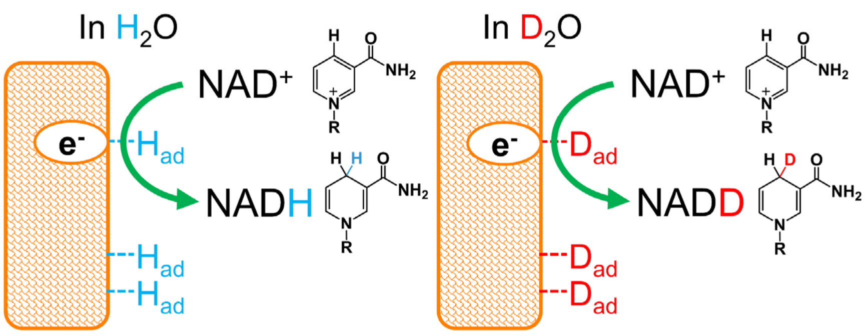

Fig. S12 The schematic description of the kinetic isotope effect (KIE) of NAD^+ reduction reaction on a Cu electrode via affecting the breakage of metal-H_{ad} bond and formation of C-H bond during the hydrogenation of the nicotinamide group.

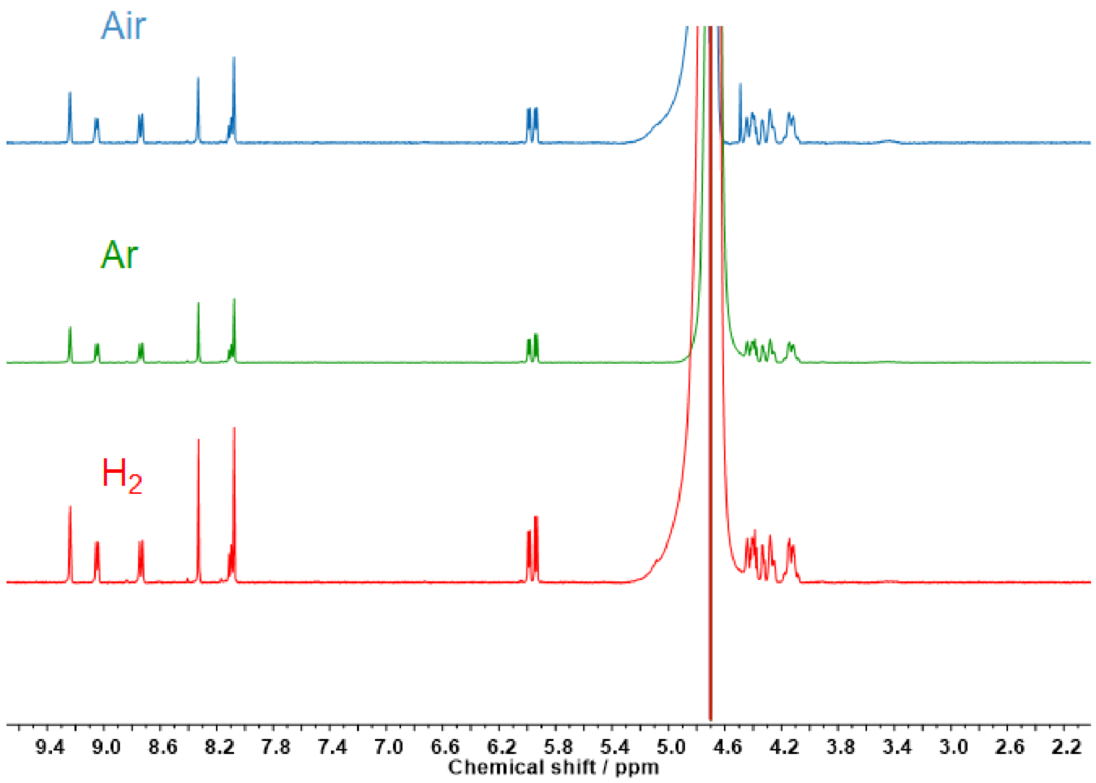

Fig. S13 The ^{1}HNMR spectrum of NAD^+ after stirring under air, Ar and H_2 atmosphere for 3 h in 0.1 M phosphate buffer (pH 7) with an NAD^+ concentration of 1 mM.

References

1. B. A. Beaupre; M. R. Hoag; J. Roman; F. H. Foersterling; G. R. Moran, *Biochemistry*, **2015**, *54*, 795-806.
2. R. W. Burnett; A. L. Underwood, *Biochemistry*, **1968**, *7*, 3328-3333.
3. R. Barin; S. Rashid-Nadimi; D. Biria; M. A. Asadollahi, *Electrochim. Acta*, **2017**, *247*, 1095-1102.
4. J. K. Noerskov; T. Bligaard; A. Logadottir; J. R. Kitchin; J. G. Chen; S. Pandelov; U. Stimming, *J. Electrochem. Soc.*, **2005**, *36*, 23-26.
5. J. Greeley; T. F. Jaramillo; J. Bonde; I. Chorkendorff; J. K. Nørskov, *Nat. Mater.*, **2006**, *5*, 909-913.
6. A. Bagger; W. Ju; A. S. Varela; P. Strasser; J. Rossmeisl, *ChemPhysChem*, **2017**, *18*, 3266-3273.
